# Radical resection of a primary unresectable duodenal cancer after chemotherapy using S-1 and cisplatin: report of a case

**DOI:** 10.1186/s40792-017-0304-4

**Published:** 2017-02-21

**Authors:** Masaru Kanehira, Yasuro Futagawa, Kenei Furukawa, Hiroaki Shiba, Tadashi Uwagawa, Katsuhiko Yanaga

**Affiliations:** Department of Surgery, The Jikei University School of Medicine, 3-25-8, Nishi-Shinbashi, Minato-ku, Tokyo, 105-8461 Japan

**Keywords:** Duodenal cancer, Chemotherapy, S-1 and cisplatin

## Abstract

**Background:**

Therapeutic outcomes and prognosis of primary unresectable duodenal cancer remains unsatisfactory, because effective chemotherapy is not established.

**Case presentation:**

A 71-year-old male diagnosed with unresectable duodenal carcinoma with distant lymph node metastases was judged inoperable (cT3N2M1 cStage in UICC^7th^). Duodenal obstruction developed due to tumor growth, and the patient underwent laparoscopic gastro-jejunostomy and then combined chemotherapy using S-1 and cisplatin. Abdominal CT revealed reduction of the tumor, and lymph node swelling almost disappeared after chemotherapy.

He underwent subtotal stomach-preserving pancreaticoduodenectomy and lymph node dissection including the para-aortic region. The final stage was fT3N1M0, StageIIIA in UICC^7th^. He developed pancreatic fistula (ISGPF grade B), which subsided, and he was discharged 29 days after operation. He underwent adjuvant chemotherapy using S-1 for 1 year, and he remains well without recurrence.

**Conclusions:**

S-1/cisplatin combination chemotherapy allowed R0 resection for advanced duodenal cancer.

## Background

Surgical resection is the only potentially cure treatment for advanced duodenal cancer. However, approximately 25% of advanced duodenal cancer cases are unresectable at the time of diagnosis [1]. Therapeutic outcomes and prognosis of primary unresectable duodenal cancer remains unsatisfactory, because effective chemotherapy is not established. We herein report a case of successful radical resection of an initially unresectable duodenal cancer that became operable after chemotherapy using S-1 and cisplatin.

## Case presentation

**Fig. 1:**
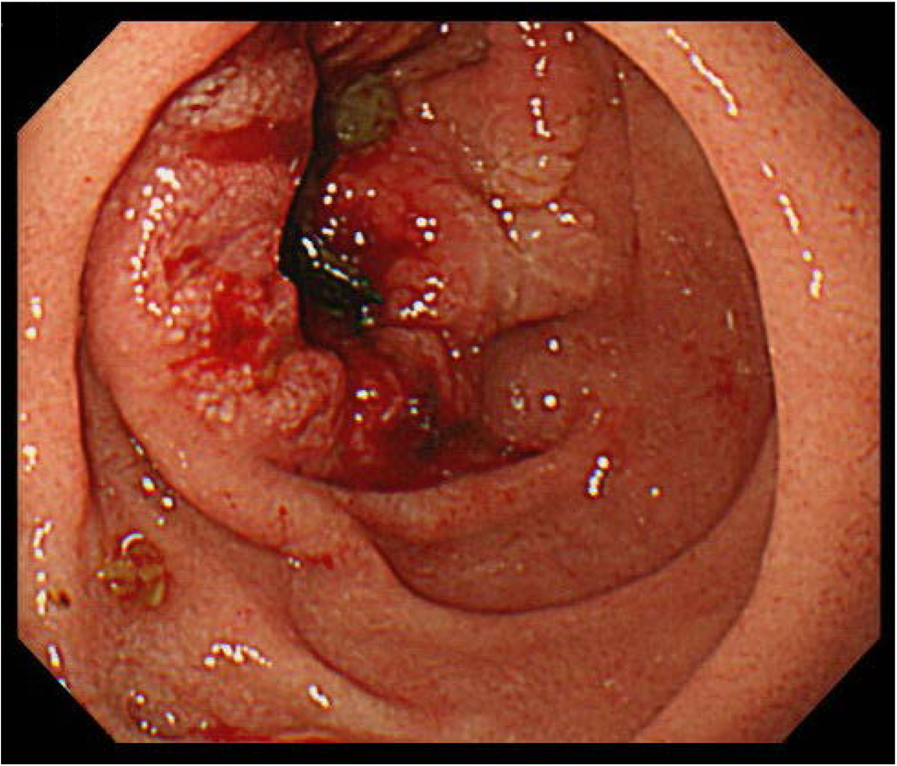
Upper gastrointestinal endoscopy revealed advanced primary duodenal cancer located in the second portion of the duodenum

**Fig. 2:**
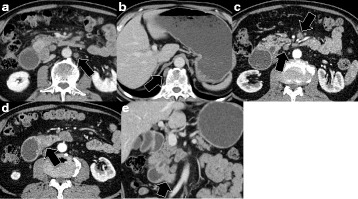
Enlarged para-aortic (**a**, *arrow*) and paravertebral lymph node (**b**, *arrow*), regional lymph nodes (**c**, *arrows*), and primary duodenal tumor (**d**, **e**, *arrows*) are detected by enhanced computed tomography

**Fig. 3:**
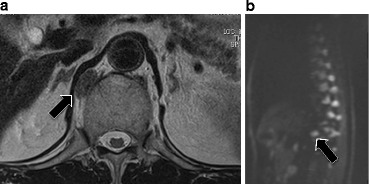
Paravertebral lymph node (**a**, *arrow*) is detected by MRI in T2. Paravertebral lymph node is high intensity in DWI (**b**, *arrow*)

**Fig. 4:**
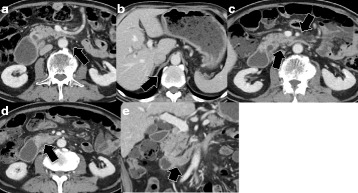
After three courses of chemotherapy, enhanced computed tomography revealed a decrease in the sizes of the para-aortic (**a**, *arrow*) and paravertebral lymph node (**b**, *arrow*), regional lymph nodes (**c**, *arrows*), and primary duodenal tumor (**d**, **e**, *arrows*)

**Fig. 5:**
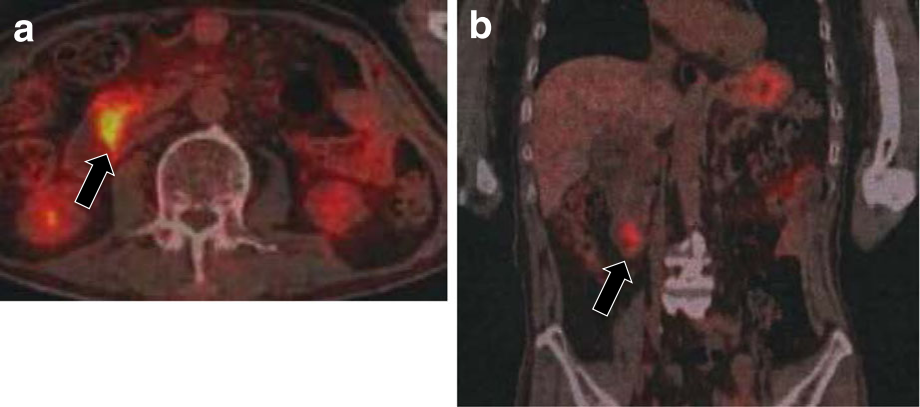
A 2-[^18^F]-fluoro-2-deoxy-d-glucose positron emission computed tomography revealed accumulation only at the site of primary duodenal tumor (**a**, **b**, *arrows*)

A 71-year-old male visited our hospital for upper abdominal fullness. Upper gastrointestinal endoscopy revealed advanced primary duodenal cancer located in the second portion of the duodenum. Duodenal cavity was closely obstructed due to the tumor, and endoscopic observation could not be performed through the tumor (Fig. 1). Enhanced computed tomography (CT) revealed a 45-mm primary duodenal tumor and enlarged regional lymph nodes. Enlarged para-aortic and paravertebral lymph nodes were also detected (Fig. 2). Elongated, soft tissue mass judged paravertebral lymph node was identified by MRI in T2 and DWI, but there is no evidence to show that this is metastatic (Fig. 3). A clinical diagnosis was primary unresectable advanced duodenal cancer with distant lymph node metastases (cT_3_, cN_2_, cM_1_ cStage IV in UICC^7th^). The patient underwent laparoscopic gastro-jejunostomy to receive bowel obstruction to allow dietary intake before chemotherapy. The patient received the combined chemotherapy using S-1 (80 mg/m^2^ per oral from day 1 to 21) and cisplatin (CDDP; 60 mg/m^2^ intravenously on day 8). This regimen was repeated at 35-day intervals. The patient had adverse events of grade 1 anorexia and diarrhea, but improved conservatively. After three courses of chemotherapy, enhanced CT revealed a decrease in the sizes of the duodenal tumor, para-aortic and paravertebral and regional lymph nodes. We judged that tumor response was stable disease (RECISTver1.1), because the size of primary duodenal tumor was slightly decreased (Fig. 4). A 2-[^18^F]-fluoro-2-deoxy-d-glucose (FDG) positron emission CT (FDG-PETCT) revealed the FDG accumulation only at the site of primary duodenal tumor (Fig. 5).

**Fig. 6:**
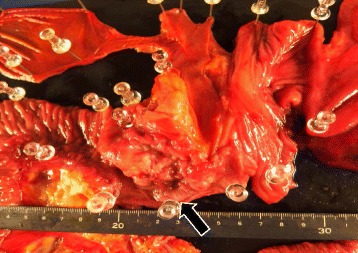
The duodenal cavity was completely obstructed by type 2 tumor located in the second portion of the duodenum (55 × 45 × 10 mm) (*arrow*). Infiltration to the ampulla of Vater was none

Since such a condition satisfied the criteria for resectability of duodenal cancer, the patient underwent laparotomy. Intraoperative frozen section was negative for microscopic para-aortic lymph node, and paravertebral lymph node could not be identified. The patient underwent subtotal stomach-preserving pancreaticoduodenectomy and lymph node dissection (No.6, 8a, 8p, 12a, 12p, 12b, 14p, 14d, 15, 16). In the resected specimen, duodenal cavity was completely obstructed by type 2 tumor located in the second portion of the duodenum (55 × 45 × 10 mm) (Fig. 6).

**Fig. 7:**
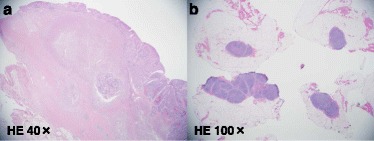
Distended changes and hydropic degeneration of nucleus were observed (**a**). Cancer cells were not detected in the no. 14 and 16 lymph nodes (**b**)

The pathological diagnosis showed adenocarcinoma infiltrating to the muscularis propria. Distended changes and hydropic degeneration of nucleus were observed, and cancer cells were not detected in the No.14 and 16 lymph nodes. These pathological changes were considered to be induced by chemotherapy, and the pathological grade was determined to be grade 2a (Fig. 7). Final stage was IIIA in UICC^7th^ (fT3N1M0). The patient suffered from postoperative pancreatic fistula grade B by the international study group of pancreatic fistula [2], but made a satisfactory recovery and was discharged on the 29th postoperative day. The patient received adjuvant chemotherapy using S-1 for 1 year and remains well for 1 year without recurrence after operation.

## Discussion

Primary duodenal cancer is a rare tumor with a poorly defined natural history, which represents 0.3 ~ 1% of all gastrointestinal tumors and 25 ~ 35% of malignant tumors of the small intestine [3]. Aaron et al. reported that the overall survival rate with duodenal cancer after radical resection at 1, 2, and 3 years were 70.0, 53.3, and 33.3%, respectively [4]. Faisal et al. reported in multivariate analysis that nodal metastasis (*p* = 0.002), advanced tumor stage (*p* < 0.001), and microscopically residual tumor (*p* = 0.02) had significant negative impacts on survival [5].

We consider that advanced duodenal cancer with lymph node metastases was poor prognosis. In this case, we chose primary chemotherapy first after bypass surgery, because several investigators reported therapeutic response for preoperative chemotherapy predicts patients’ prognosis after radical resection [6]. Conversion surgery has a risk of morbidity and mortality in addition to decrease of QOL due to surgery. Because pathological complete response by chemotherapy is rare in gastrointestinal malignancies, we chose radical resection when para-aortic lymph node metastasis disappeared [7].

The feasibility of chemotherapy and its regimens for primary duodenal cancer are still controversial. In Japan, combined chemotherapy using S-1 and cisplatin is a standard treatment for advanced gastric cancer [8]. However, to the best of our knowledge, only few cases of conversion from primarily unresectable duodenal cancer to resection by chemotherapy using S-1 and cisplatin have been reported (Table 1). In this case, we chose combined chemotherapy using S-1 and cisplatin following the regimen of advanced gastric cancer, because chemotherapy for duodenal cancer is not established. On the other hand, Edwin et al. recommend for primary unresectable duodenal adenocarcinoma the chemotherapy regimens for colorectal cancer, such as leucovorin and 5-fluorouracil with irinotecan (FOLFIRI) or oxaliplatin (FOLFOX) [1]. Wang et al. reported the clinically complete response of advanced duodenal adenocarcinoma by chemotherapy using oxaliplatin and S-1 [9]. In addition, Chris et al. reported clinical significance of radical resection with chemo-radiation therapy (CRT) in overall survival rate as compared to surgery alone (83% for surgery with CRT versus 53% for surgery alone, *p* = 0.07) [10].

**Table 1:** Reported cases of duodenal cancer anaplastic preoperative chemotherapy using S-1 and cisplatin in Japan

	Author	Year	Age/sex	LN metastases	Location (duodenum)	Effectiveness	Operation	Prognosis
1	Egawa [13]	2008	60/M	Periduodenal	2nd portion	PR	PD	Alive, 6 months
2	Kang [14]	2009	48/M	Supraclavicular LN Para-aortic LN	4th portion	PR	Partial resection	Died, 15 months
3	Mima [15]	2011	53/F	None	2nd portion	PR	PD	Alive, 12 months
4	Our case		71/M	Para-aortic LN	2nd portion	SD	PD	Alive, 12 months

*PD* pancreaticodoudenectomy, *PR* partial response, *SD* stable disease

Adjuvant chemotherapy for primary duodenal cancer is also not established.

Because of microscopic curative resection, we chose S-1 alone as adjuvant chemotherapy for prevention of cancer recurrence following the regimen of gastric cancer [11].

Several investigators reported usefulness of PET-CT on assessment of therapeutic efficacy in patients with distant metastasis [12]. Our case did not take PET-CT before chemotherapy, but PET-CT before chemotherapy may help for the assessment of chemotherapeutic response.

## Conclusions

From our experience, S-1/cisplatin combination chemotherapy allowed R0 resection for advanced duodenal cancer. Previous reports suggested that multidisciplinary therapy including surgery, chemotherapy, and radiotherapy may be required to improve therapeutic outcome of advanced duodenal cancer.

## Abbreviations

CRT: Chemo-radiation therapy
FDG-PETCT: 2-[^18^F]-fluoro-2-deoxy-d-glucose positron emission CT
